# Time warping of evolutionary distant temporal gene expression data based on noise suppression

**DOI:** 10.1186/1471-2105-10-353

**Published:** 2009-10-26

**Authors:** Yury Goltsev, Dmitri Papatsenko

**Affiliations:** 1 Department of Molecular and Cell biology, University of California, Berkeley, USA

## Abstract

**Background:**

Comparative analysis of genome wide temporal gene expression data has a broad potential area of application, including evolutionary biology, developmental biology, and medicine. However, at large evolutionary distances, the construction of global alignments and the consequent comparison of the time-series data are difficult. The main reason is the accumulation of variability in expression profiles of orthologous genes, in the course of evolution.

**Results:**

We applied Pearson distance matrices, in combination with other noise-suppression techniques and data filtering to improve alignments. This novel framework enhanced the capacity to capture the similarities between the temporal gene expression datasets separated by large evolutionary distances. We aligned and compared the temporal gene expression data in budding (*Saccharomyces cerevisiae*) and fission (*Schizosaccharomyces pombe*) yeast, which are separated by more then ~400 myr of evolution. We found that the global alignment (time warping) properly matched the duration of cell cycle phases in these distant organisms, which was measured in prior studies. At the same time, when applied to individual ortholog pairs, this alignment procedure revealed groups of genes with distinct alignments, different from the global alignment.

**Conclusion:**

Our alignment-based predictions of differences in the cell cycle phases between the two yeast species were in a good agreement with the existing data, thus supporting the computational strategy adopted in this study. We propose that the existence of the alternative alignments, specific to distinct groups of genes, suggests presence of different synchronization modes between the two organisms and possible functional decoupling of particular physiological gene networks in the course of evolution.

## Background

Comparative analysis of evolutionary changes in distant organisms at the level of gene expression requires cross-matching (alignment) of temporal microarray data covering developmental time courses or cell cycles. Alignment of time series data or time warping allows side by side comparison of orthologous gene expression on a relative time scale [1-5]. The time warping produces non-linear alignment paths, which help estimate the relative duration of similar steps in the life cycle of diverged species. In addition, aligned temporal datasets can reveal concordantly and discordantly expressed pairs of orthologous genes or groups of genes. Currently available time-warping algorithms [6] stem out from early methods of speech recognition [7]. Benchmarking tests show that the existing methods under perform on noisy datasets and require accommodation to temporal expression data from organisms separated by large evolutionary distances [see Additional file 1 - Figures S1-S3] (and at UC Berkeley online resource: ).

Here, we tested several noise suppression techniques, in order to optimize global alignment between the time series data from two species, separated by ~400 million years of evolution, budding (*Saccharomyces cerevisiae*) and fission (*Schizosaccharomyces pombe*) yeasts. Traditionally, yeast cell cycle served as a model system to study regulation of the periodic gene expression, replication, and cell division [8,9]. Evolution of the periodic gene expression in yeast has been explored based on classification approaches using temporal gene expression data [10-12], where an individual periodicity score was assigned to each ortholog and these periodicities and phases were then compared. In contrast to the classification approach, time warping captures information from all orthologous profiles in a single test.

Exploration of alignments, constructed for *S. cerevisiae *and *S. pombe *using available methods and programs [1,6] [see Additional file 1 - Figures S1-S3] (and the UC Berkeley online resource) revealed presence of long gaps and noisy alignment paths. In this study, we introduced and thoroughly tested a novel data treatment and alignment framework, based on noise-suppression methods and elements of Kruscal-Liberman alignment algorithm [6]. The framework allowed us to override interspecific noise and to construct a global alignment for the two yeast species.

This global alignment supported previously observed differences in duration of G1 and G2 cell cycle phases [13]. In order to explore alternative alignments, the pairs of the orthologous expression profiles have been aligned individually and the resulting individual alignment paths were clustered using common clustering algorithms [14]. Using this approach, we found gene groups or "time clusters," in which the relative synchronization modes (alignment paths, characteristic to each time cluster) were different from the global alignment path. Our analysis suggested that evolutionary shifts in durations of G1/G2 cell cycle phases are manifested in the expression timing of replication machinery and ribosomal genes. Instead, gene expression in mitochondria was desynchronized or evolutionary "disconnected" from the replication and housekeeping genes due to high autonomy of that organelle.

## Results and discussion

### Data selection and noise removal

Success of the cross-specific time warping critically depends on the level of noise in time series expression data, "internal noise" and on the evolutionary variability in the gene expression between the two species or "external noise". The internal noise appears, for instance, due to the measurement errors between different microarrays (time points) and due to desynchronization of cell culture over time; the external noise is the result of accumulated in evolution differences in the orthologous expression or differences in expression caused by experimental conditions, such as selection of cell culture synchronization method. In this perspective, problems connected with the alignment construction are largely problems related to noise reduction and noise overriding.

Ability to judge quality of alignments critically depends on the input data; data selection helps to find the least noisy/most reliable datasets. Therefore all 70 pairwise combinations of publicly available *S. cerevisiae *and *S. pombe *datasets [10-13,15-17] were explored using Kruscal-Liberman algorithm [6] based on either Euclidean or Pearson distance matrices (see Methods and UC Berkeley online resource).

We adopted Pearson distance matrices to produce highly informative comparisons between time series and to cope with the external (evolutionary) noise (see Methods). The distance matrices revealed discernible periodic patterns similar to that observed in simulated periodic datasets [see Additional file 1 - Figure S1]. Notably, alignments based on the Pearson distance matrices sustained much higher external noise and were capable of capturing even subtle similarities between orthologous datasets [see Additional file 1 - Figures S1-S3] (and the matrices for all 72 pairwise comparisons, available from UC Berkeley web resource). Judged by the quality of the observed periodic patterns, we have selected for detailed analysis two pairs of datasets: *S. cerevisiae *synchronized by *α*-factor [12], and *S. pombe *synchronized either using *cdc25 *temperature sensitive mutant or elutriation [17].

Based on the amplitude of gene expression in the course of the life cycle, all genes in the yeast genome can be conditionally separated into (*i*) cell-cycle dependent (oscillating) (*ii*) constitutively expressed and (*iii*) inducible (not expressed or expressed constitutively in our datasets). Low oscillating and constitutive genes contribute less or no information to the global alignment, moreover, their actual expression dynamics can be masked by the internal noise. Therefore, we removed the low-variant genes from the selected datasets to improve sensitivity of the method. In the prior studies [10,17], Fourier analysis was used to eliminate the low-cycling genes. However, several factors, such as biased contribution of synchronization approaches, short duration of the datasets (two cell cycles) and high internal noise can make Fourier analysis fail for many genes, which, in fact, do cycle significantly. In the case of the yeast cell cycle, biological replicates were not available, and standard ANOVA-based filtering could not be applied. Therefore, we designed SNR (Signal-to-Noise Ratio) filter to eliminate noisy and low-cycling profiles. The SNR filter is analogous to ANOVA, but requires no replicates [see Additional file 1 - Figures S4, S5] (and Methods section). Statistical model for SNR takes into account assumption that periodically expressed genes would gradually increase or decrease expression level from one time point to another. In mathematical terms, the variance of point-to-point changes in a gene expression profile should be less than the variance of the data itself.

The two selected datasets were filtered using the SNR method to remove the low-variance profiles and smoothed using Gaussian method to minimize the internal noise (see Methods section). The SNR filtering reduced the initial number of the orthologous profile pairs in the selected datasets (*α*-factor - *S. cerevisiae *vs. *cdc25 *- *S. pombe*) to 3193 or otherwise to 2518 genes in *S. pombe *and 2169 genes in *S. cerevisiae *(see ortholog matching in Methods). An apparent contradiction between the previously reported number of cycling genes (500) [18] and the number of genes investigated in this study (>2000) is explained by the fact that all significantly changing profiles (high variance) were scored in our study, even if they displayed moderate Fourier power at the desired period [see Additional file 1 - Figure S5]. We believe that in most instances low Fourier scores reflected biased expression at the start of the cell culture synchronization, desynchronization of cells after several cycles and/or measurement errors. After removal of the previously reported best cycling 500 profiles from our dataset, the distance matrix and the alignment did not change significantly [see Additional file 1 - Figure S6]. This test provided evidence that periodicity is present in the additional genes, as compared to prior studies, although it might be masked by the noise or conditions of the culture synchronization. We also found that Gaussian smoothing significantly improved detection of periodicity in the expression profiles (data not shown).

### Time warping results

**Figure 1:**
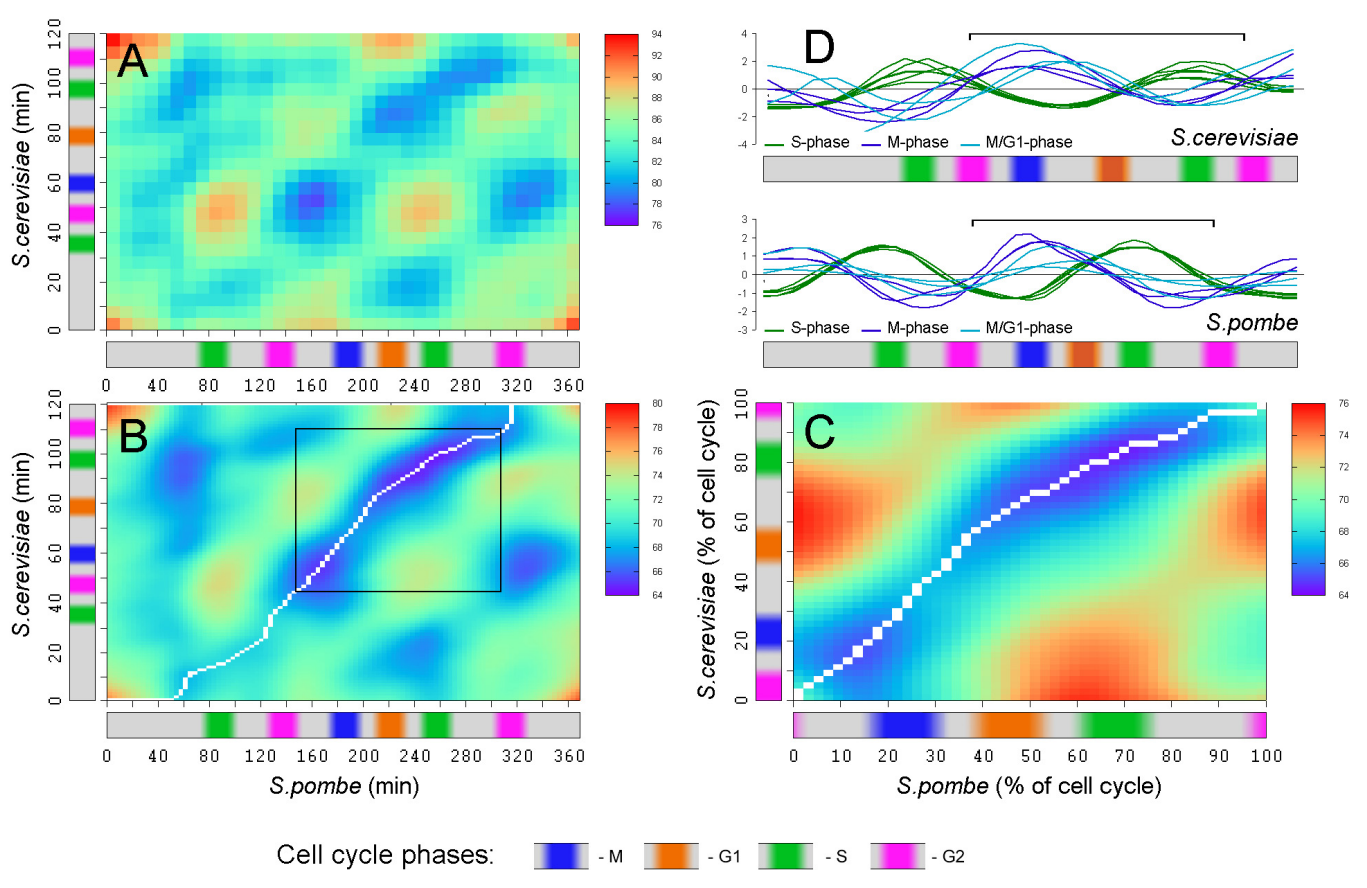
**Time warping of a single cell cycle**. (A-C) Pearson distance matrices constructed for *S. cerevisiae *- *S. pombe *microarray time series data. *S. cerevisiae *cells were synchronized using *α*-factor [12]*S. pombe *cells were synchronized using *cdc25 *temperature sensitive mutant [17]. Periodically arranged patterns mark similarities in the gene expression; bars on the left and the bottom mark cell cycle stages in both species. (A) Raw data, before treatment. (B) Smoothed and filtered data, white line indicates global alignment path, black box marks data range corresponding to a single cell cycle in both species. (C) Similarity matrix for the data range marked by black box in (B), white line indicates the global alignment path. (D) Cell cycle phases were established using standard set of markers, such as histones (S-phase), *CDC20*, *CDCD5 *(M-phase), *DBF2*, *CDC6 *(M/G1-phase) [17]. Brackets show ranges corresponding to a single cell cycle.

Distance matrices for the full datasets, each spanning approximately 2 cell cycles, were inspected to identify data ranges, corresponding to a single cell cycle in each species (see Figure 1B). A global alignment path has been constructed using time warping based on Kruscal-Liberman algorithm [6] using Pearson distance matrices (Figure 1A, B). Variations in the data treatment and the Pearson matrix construction parameters produced several possible paths; nevertheless, all successful alignments (gapless or with minimal gaps) followed nearly identical paths [see Additional file 1 - Figure S7].

**Figure 2:**
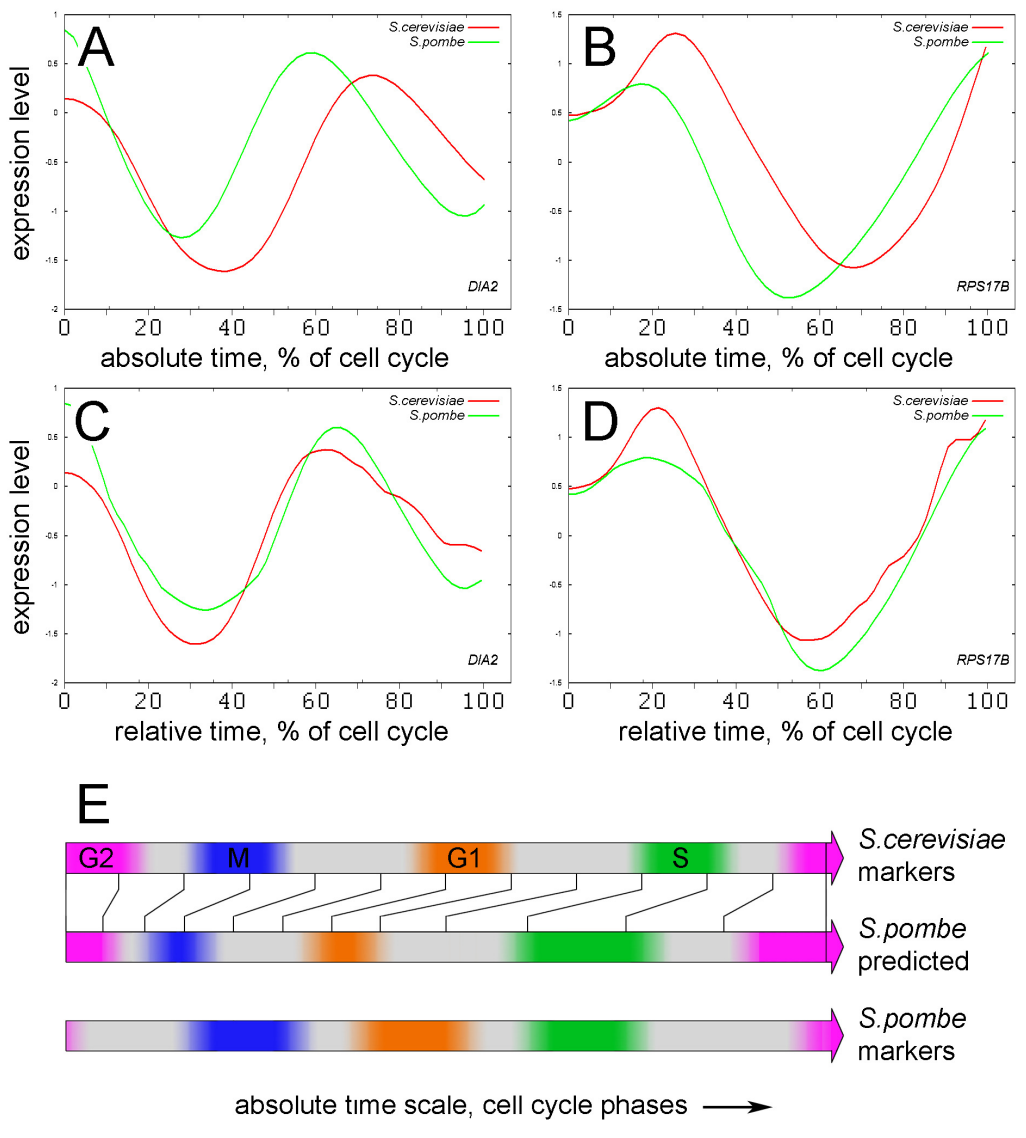
**Time warping and concordantly expressed genes**. (A, B) Orthologous expression profiles for genes from the two yeast species are superimposed on the absolute timescale, before alignment. The selected data ranges correspond to ranges marked in Figure 1B. (C, D) Profiles for the same genes, adjusted according to the global alignment path, (relative time scale). (E) Correspondence between the cell cycle phases in *S. cerevisiae *and *S. pombe*, established based on the global alignment. Notice collapse of M-G1 phase and expansion of S-G2 phase region in *S. pombe*.

Data ranges, corresponding to a single cell cycle were selected based on the periodic patterns observed on the distance matrix and standard cell cycle markers, characteristic to specific phases of the cell cycle [10,17]. The selected data ranges were aligned according to the global alignment path shown in the Figure 1C. Time warping for a single cell cycle has clearly shown that the comparative duration of cell cycle phases in the two species is different (see Figure 2E) and it is in a good agreement with the existing data [13]. According to the global alignment path, *S. pombe *has longer G2 phase and shorter G1 phase. This result supported our computational strategy, selected with respect to the high divergence between the analyzed species.

**Figure 3:**
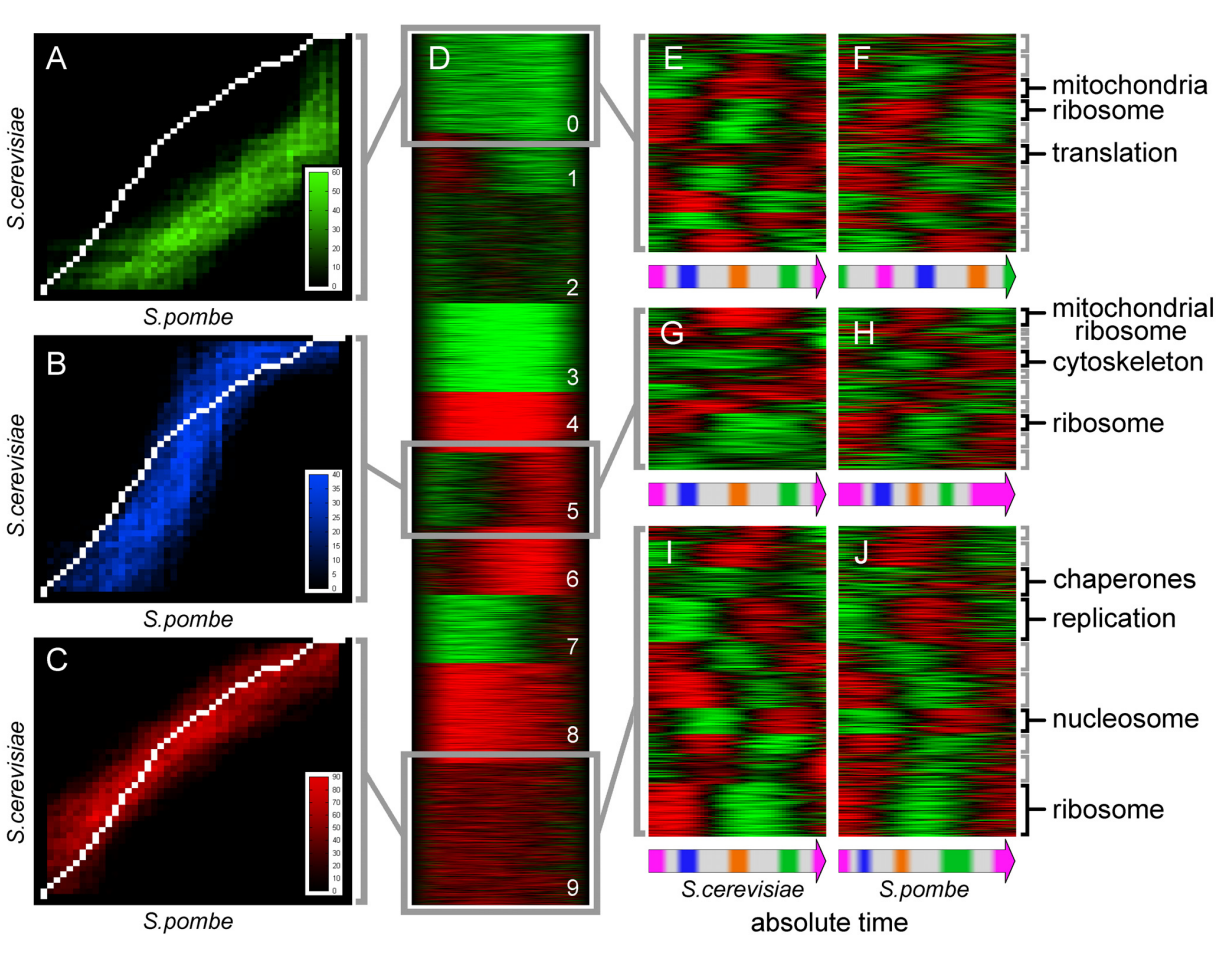
**Heterochrony of gene expression**. (A-C) Examples of clusters of individual alignment paths (red, blue and green - different time clusters) in comparison with the global alignment path (white line). Color intensity on the histograms indicates number of individual alignment paths crossing specific matrix locations. (D) All individual alignment paths clustered using Cluster3 software. Grey boxes indicate clusters selected for matrix representation on A, B and C. (E) Results of consequent clustering of *S. cerevisiae *expression profiles, corresponding to genes present in the time cluster 0 (panels A, D). (F) *S. pombe *orthologs corresponding to the profiles in E. (G) Results of consequent clustering of *S. cerevisiae *expression profiles, corresponding to genes present in the time cluster 5. (H) *S. pombe *orthologs corresponding to the profiles in G. (I) Results of consequent clustering of *S. cerevisiae *expression profiles, corresponding to genes present in the time cluster 9. (J) *S. pombe *orthologs corresponding to the profiles in I. Timelines below the panels E-J demonstrate alternative phase shifts in the expression of *S. pombe *orthologs. Functionally related genes are marked with black brackets on the right side.

Inspection of orthologous profile pairs in the aligned datasets revealed instances of both concordantly and discordantly expressed genes. 518 expression profiles, corresponding to approximately ~400 genes had very similar or nearly identical expression phasing in both organisms. Figure 2 shows the expression profiles of two orthologous pairs, which appear discordant on the absolute timescale (unaligned) and are nearly identical on the relative timescale, after time warping. *DIA2 *(Figure 2A, C) is the origin-binding F-box protein that plays a critical role in DNA replication and maintaining genome integrity. *S. cerevisiae *strains with *DIA2 *deletions have a high rate of endogenous DNA damage and are defective in S-phase progression [19,20]. Gene *pof3*, the ortholog of *DIA2 *in *S. pombe *has a similar function. In *pof3 *mutants the telomeres are substantially shortened and the normal telomere transcription/silencing is disrupted [21]. Another example, gene *RPS17B *(Figure 2B, D) encodes the ribosomal protein 51 (*rp51*) of the small (40S) subunit and displays concordant expression in the unaligned data sets as well. However, after time warping, the ribosomal protein has identical expression phasing in both yeast species. The majority of other ribosomal genes and genes involved into the ribosome biogenesis also displayed identical phasing of expression in the aligned datasets [see Additional file 1 - Figure S8] (and Figure 3).

### Alternative alignment paths and heterochrony

Along with the global alignment, alignments for each individual pair of orthologs from the selected datasets were constructed and explored as well. This analysis has shown that the majority of concordantly expressed orthologous genes produced pairwise alignment paths similar to the global one (see examples in Figure 2 and Figure 3C). At the same time, individual alignments of discordantly expressed ortholog pairs often produced alignment paths different from the global (Figure 3A, B). We explored the pairwise alignments to see whether there are alternative alignment paths, common to certain groups of genes expressed discordantly with respect to the global alignment (idea first proposed by J. Aach and G. Church [1]).

In order to reveal the alternative paths of time warping, all ortholog pairs were aligned one by one, using the Gene-warp program and the resulting individual alignment paths were clustered using k-means clustering with arbitrarily selected number of clusters *k *= 10 [14] (see Methods section). Figure 3 shows "time clusters" produced by the clustering of individual alignment paths. Technically, each time cluster corresponds to a group of individual profile pairs with similar pairwise time warping. From the biological standpoint, the time clusters correspond to synchronization groups; within each group, the expression synchrony among all genes is maintained during evolution, while synchrony between the groups (time clusters) is apparently lost (see Figure 3A-C).

The concordantly expressed genes (regardless of the phasing of their expression) formed the largest time cluster or the largest synchronization group (Figure 3C, D), containing 518 expression profiles. As expected, the average alignment path of this largest synchronization cluster was close to the global alignment path. Other time clusters revealed different levels of desynchronization with the global path, varying from moderate (see cluster 8 in Figure 3D) to extreme (clusters 3 and 4 in Figure 3D).

To explore why certain gene groups maintained synchronization in evolution, expression data, composing individual time clusters, have been explored further in *S. cerevisiae *dataset by consequent clustering of the expression profiles and GO-terms enrichment analysis. It has been found that the largest *S. cerevisiae *time cluster (Figure 3C) matched the global alignment path and contained expression profiles for many ribosomal and replication-related genes (Figure 3I, J) [see also Additional file 1 - Table S1]. Synchronization between the replication machinery, ribosomal and housekeeping genes suggests coordination between the cell division and the cell growth [22] in both yeast species.

We also inspected the composition of the time clusters deviating from the global alignment path (Figure 3D, clusters 0 and 5). According to the results of the GO-term enrichment assays, some of these time clusters contained genes involved in respiration and protein synthesis in mitochondria (see Figure 3E-H). Such desynchronization or heterochrony observed between the mitochondrial and the ribosomal/replication genes can be attributed to the semi-autonomy of mitochondria and the mitochondrial gene expression [23]. Mitochondrial biogenesis in *S. pombe *is more similar to higher eukaryotes [24], so it is quite possible that the independent synchronization of some mitochondrial genes is maintained in higher eukaryotes as well.

According to endosymbiotic theory [25], mitochondria entered eukaryotic cells nearly a billion years ago. Apparently, since then, some of the mitochondrial pathways (respiration, ribosome biogenesis) maintained their own, internal, synchronization of gene expression. Phase shift in expression of ribosomal and mitochondrial ribosomal genes detected in this study (see Figure 3G-J) appears to support hypothesis of decoupling of oxidative and reductive biochemical pathways in the yeast cell cycle [26,27], and possibly represents an example of heterochrony [28] at the level of gene expression.

Time clustering, combined with the consequent profile clustering, helps in the superimposing and interpretation the evolutionary changes in gene expression. Without time clustering [see Additional file 1 - Figure S9], superposition of the orthologous profiles is much less informative (compare Figure 3E-J with Figure S9 D, E).

## Conclusion

### Desynchronization of gene expression in evolution

Data selection/filtering and noise suppression strategy made it possible to build a global alignment between very diverse temporal expression data for yeast species, separated by ~400 million years of evolution. Specifically, it has been found that the Pearson metrics in the context of Kruscal-Liberman time warping enables aligning very diverse time series data. Alignments, constructed for the yeast species have been validated using prior biological knowledge.

Recent studies in the evolutionary genomics field suggested presence of conservation between sub domains of large gene networks [29-31]. Preceding genome-wide studies of conserved genetic interactions in *S. cerevisiae *and *S. pombe *demonstrated conservation of genetic interactions between particular sets of genes, corresponding to protein complexes or pathways [32].

In this work, comparative analysis of gene expression dynamics has shown that parts of large gene networks (presumably corresponding to time-clusters) maintained substantial temporal synchrony in the course of evolution. The time warping in combination with the path and profile clustering allowed tracing synchronization for some housekeeping, structural and replication genes. However, analysis of regulatory components of cell cycle, such as cyclins, revealed no such synchronization or other shared evolutionary trends (data not shown). One possible reason for this is the dramatic rewiring of regulatory pathways during evolution.

Mathematical strategies, described in the current work, can be applied to comparative analysis of expression data in any pair of organisms, separated by hundreds of millions years of evolution. The following factors may limit the area of application: (*i*) variability in gene expression under different experimental conditions (synchronization method, [see Additional file 1 - Figure S10 and Table S2]; (*ii*) strikingly different alternative alignment paths, specific to large group of genes (heterochrony, see Figure 3); (*iii*) distortion of gene expression profiles as the result of normalization and Gaussian smoothing.

## Methods

### Microarray data and low-level data processing

All available microarray time-series data sets for *S. cerevisiae *and *S. pombe *[10-13,15-17] were downloaded from the author's sources [33] or NCBI GEO database [34]; ortholog tables were obtained from Valerie Wood (Sanger Institute) [35]. In cases where a single gene in one species corresponded to multiple orthologs in another species, multiple profile pairs were generated by duplicating the single expression profile and superimposing it to all matching orthologs. Alignment of data, obtained using different methods of cell synchronization has shown that there is 50-80% of consistency between dataset pairs displaying clear periodic patterns [see Additional file 1 - Figure S10 and Table S2].

Low-level data processing included the following steps: signal-to-noise filtering (SNR), upsampling, Gaussian smoothing and Z-score normalization. Z-score normalization was done using standard methods [36]. Upsampling and Gaussian smoothing were performed in order to reduce noise and improve quality of alignments. Upsampling is a standard way of converting analog signals to digital, new sample rate should be at least 2× higher than the highest frequency in the original signal (Nyquist-Shannon sampling theorem). Accordingly, all input datasets (37 time points maximum) were upsampled in this study to 100 time points. Gaussian filter was used to remove high frequencies, much higher than frequencies related to the cell cycle, assuming that the high frequencies are noise. Together, upsampling and Gaussian filtering might have effects similar to the interpolated time warping described earlier [1]. Attempts to filter out non-periodic profiles using Fourier methods [10] eliminated too many profiles with high variance, which is not surprising given very small number of periods in the microarray data (~2.5). Therefore, Fourier filter was replaced by signal-to-noise filter (SNR) [see Additional file 1 - Figure S1].

Original SNR filter based on non-parametric statistics has been designed for the analysis of microarray time series data, which lacks biological replicates. Consider local point-to-point variation Δ*x *between the neighboring time points *i *and *i*+1 in *j*^th ^expression profile:

(1)

If the variance , the noise is high and the profile (probe) needs to be excluded from consideration (see additional file 1). Each expression profile in every data set was scored using the following log-ratio:

(2)

In this formula, σ^2^(Δ*x*) (pseudocount) is the average variance of the point-to point variation (noise) taken for all profiles of the entire data set (see eq. S1-S6 in the additional file 1).

### Similarity matrices and Time warping

Euclidean similarity matrices take into account only the levels of gene expression at a given time point [1]. We found that this commonly used method failed in the case of alignment between *S. cerevisiae *and *S. pombe *[see Additional file 1 - Figure S2]. To improve sensitivity of time warping, we replaced Euclidean matrices by Pearson similarity matrices. Given time window size parameter *n*, {*n *∈ (2*N *+ 1)} one can compute value of the Uncentered Pearson correlation *r *for a given *k*^th ^pair of the orthologous profiles *a *and *b *at the time points *i *and *j *as follows:

(3)

This procedure returns agreement between segments of the two profiles, each of length *n *time points, centered on time point *i *and *j *correspondingly. Similarity between the time point *i *from dataset  and the time point *j *from dataset , for all *M *pairs of orthologous profiles, was computed as a standard Pearson distance:

(4)

The Pearson similarity matrices have higher sensitivity and produce better alignments [see Additional file 1 - Figure S2] (and UC Berkeley web resource) then Euclidean as they collect more information in each point-to-point comparison (see eq. S7-S12 in the additional file 1 for more details).

### GT-Warp research software package

The described method has been implemented in software package GT-Warp. The package includes the following programs and utilities: "AVF-filer" and "RZ-smooth" are programs for low-level data filtering and processing. These programs include common methods such as Fourier analysis, ANOVA, F-test, Gaussian smoothing, resampling, and normalization. AVF-filer program also includes SNR method described above and "VF-stat" utility for simulating SNR score distribution in random data. The program "Time-warp" incorporates both Euclidean and Pearson methods (see above), generates global alignment matrices using Kruscal-Liberman algorithm [6], and has graphical outputs, such as shown in Figure 1. The program "Gene-warp" incorporates the same methods and is intended for one-to-one alignment of orthologous profiles. Gene-warp produces alignment paths data, which can be clustered using standard methods, such as Cluster 3.0 [37]. GT-Warp package also includes program "M-align" for aligning datasets based on matrices produced by Time-warp and a "Prf-browser" to browse and display orthologous profiles on the same plot. GT-Warp package has been written in Perl and compiled for Win32; source code is available upon request, Win32 distribution, help, and test files are available from UC Berkeley online resource.

### Clustering alignment paths

Alignment paths for individual profile pairs were generated using Euclidean method (Gene-warp program). The paths were clustered using K-means clustering method, producing 10 temporal clusters using Cluster 3.0 program with default parameter settings [14,37]. *S. cerevisiae *expression profiles from each temporal cluster (or "time cluster") were clustered again, using K-mean clustering method, producing 10 sub-clusters within each of the 10 time clusters. Enrichment of gene ontology terms in the time clusters and subclusters was carried out using GeneMapp 2.0 package [38] [see Additional file 1 - Table S1].

## Authors' contributions

UG and DP conceived the study and designed the research strategy. UG prepared yeast datasets for the analysis; DP developed algorithms, software and carried out computations. UG and DP analyzed results and wrote the manuscript. All authors read and approved the final manuscript.

## Supplementary Material

Additional file 1 **Time warping of temporal gene expression data: algorithms and controls**. The file contains details for signal to noise ratio filtering (SNR) algorithm, time-warping algorithm, results of benchmarking versus existing methods and programs, and assessment of program parameters. Click here for file
