# Supplementary material for: Time warping of evolutionary distant temporal gene expression data based on noise suppression
Source: BMC Bioinformatics. 2009 Oct 26;10:353. doi: 10.1186/1471-2105-10-353 (PMC2771023; doi:10.1186/1471-2105-10-353)
Supplement: Additional file 1 — Time warping of temporal gene expression data: algorithms and controls. The file contains details for signal to noise ratio filtering (SNR) algorithm, time-warping algorithm, results of benchmarking versus existing methods and programs, and assessment of program parameters. [file 1471-2105-10-353-S1.PDF]

Time warping of evolutionary distant temporal gene expression data based on noise suppression

Supplementary data

Yury Goltsev and Dmitri Papatsenko*
Department of Molecular and Cell Biology,
University of California, Berkeley.
*E-mail: dxp@berkeley.edu

Signal to noise ratio (SNR) filtering

Let us consider local point-to-point variation of a variable X, representing time points of a gene expression profile:

$$\Delta x_i = \frac{x_{i+1} - x_i}{2} \quad \text{(S1)}$$

Variance of this function is equal to:

$$\sigma^2(\Delta x) = \frac{1}{(N-1)} \sum_{i=1}^{N-1} \left(\frac{x_{i+1} - x_i}{2} - \overline{\Delta x} \right)^2 \quad \text{(S2)}$$

However, for a random and infinite X, $\overline{\Delta x}$ =0 (uncentered). This is also true for the periodic expression profiles. So the eq S2 becomes:

$$\sigma^2(\Delta x) = \frac{1}{(N-1)} \sum_{i=1}^{N-1} \left(\frac{x_{i+1} - x_i}{2} \right)^2 \quad \text{(S3)}$$

Consider variance of the X itself:

$$\sigma^2(x) = \frac{1}{N} \sum_{i=1}^{N} (x_i - \bar{x})^2 \quad \text{(S4)}$$

If variance $\sigma^2(x)$ for a given expression profile is significantly higher than the variance $\sigma^2(\Delta x)$, then noise in the profile is low. This statement assumes that gene expression changes gradually from point-to-point and that the measurements should reflect this property, unless the sampling rate is too low. Indeed, this is the case for most of the “ideal” expression profiles, considered in this study (see the **Figure S1**). One possible way to account for the noise level is to consider log-ratio of the two variances S for each expression profile:

$$S = \log\left(\frac{\sigma^2(x)/2 + \overline{\sigma^2(\Delta x)}}{\sigma^2(\Delta x) + \overline{\sigma^2(\Delta x)}} \right) \quad \text{(S5)}$$

Here $\overline{\sigma^2(\Delta x)}$ (pseudocount) is the average variance of Δx, taken across all M data sets for the entire microarray:

$$\overline{\sigma^2(\Delta x)} = \frac{1}{M}\sum_{j=1}^{M}\sigma_j^2(\Delta x) \quad \text{(S6)}$$

It is easy to see that for a large enough set of random values, the ratio in eq S5 becomes 0. In order to determine cutoff value of *S*, 10^6 profiles, containing random values only were scored using formula eq. S5. This produced a null model (null distribution) for *S* (see **Figure S1G**). Based on the models, 90% level of significance was selected to eliminate noisy profiles from *S.cerevisiae* and *S.pombe* expression data. After this procedure, 3193 out of 4441 orthologous profile pairs remained (72%).

Alignment algorithm and construction of distance matrices

Classic time-warping algorithm (Kruskal-Liberman) [1-3] [4] [5] [6] inductively computes alignment scores $D_{i,j}$ during forward matrix filling step. The algorithm takes as an input a matrix of pairwise distances *d* (computed as below). The first row and the first column of the alignment score matrix are filled as follows:

$$D_{1,1} = 0, D_{i+1,1} = D_{i,1} + \frac{\tau}{2}d\left(\vec{a_i},\vec{b_1}\right), D_{1,j+1} = D_{1,j} + \frac{\mu}{2}d\left(\vec{a_1},\vec{b_j}\right) \quad \text{(S7)}$$

Di,j for $1 < i \leq n$, $1 < j \leq m$ is inductively defined as follows:

$$D_{i,j} = \min\begin{cases} D_{i-1,j-1} + \frac{\tau+\mu}{2}d\left(\vec{a_i},\vec{b_j}\right) \\ D_{i-1,j} + \frac{\tau}{2}d\left(\vec{a_i},\vec{b_j}\right) \\ D_{i,j-1} + \frac{\mu}{2}d\left(\vec{a_i},\vec{b_j}\right) \end{cases} \quad \text{(S8)}$$

In the formula (S7) τ and μ are the corresponding time intervals or time cost coefficients for the data points in the first and in the second datasets. $d\left(\vec{a_i},\vec{b_j}\right)$ is the similarity between the two vectors, each representing expression of the same set of *M* genes in the corresponding time point *i* and *j* or several time points related to *i* and *j*. The optimal path (optimal alignment) through the filled matrix is identified using trace back procedure, identical to that in the case of alignment of biological sequences.

Alignment score *S*(*A*) for a time warping *A* is the sum taken over all scores $D_{i,j}$, belonging to the alignment path of *A*:

$$S(A) = \sum_{i,j \in A} D_{i,j} \quad \text{(S9)}$$

In one case, the distances $d\left(\vec{a_i},\vec{b_j}\right)$ were computed as Euclidean distances Velichko, 1970 #15}[2, 3]:

$$d_E\left(\overrightarrow{X_i^a},\overrightarrow{X_j^b}\right)=\sqrt{\sum_{k=1}^{M}\left(x_{ik}^a-x_{jk}^b\right)^2}$$
$$\overrightarrow{X_i^a}=(x_{i1}^a,x_{i2}^a,x_{i3}^a,\ldots,x_{iM}^a) \qquad \text{(S10)}$$

In another case, a parameter n, $\{n \in (2N+1)\}$, or time window was introduced, defining how many time points to take into account for each a_i <–> b_j comparison. This similarity measure (now n time points in one data set versus n time points in another data set) is the uncentered Pearson correlation r, computed over the set of profile pairs M:

$$d_P\left(\overrightarrow{X_i^a},\overrightarrow{X_j^b}\right)=\sqrt{\sum_{k=1}^{M}\left(1-r^{ab}(i,j,k,n)\right)^2} \qquad \text{(S11)}$$

Given time the window parameter n, one can compute value of the Uncentered Pearson correlation r for a given pair k of the orthologous genes as follows:

$$r^{ab}(i,j,k,n)=\frac{1}{n}\sum_{l=-(n-1)/2}^{l=(n-1)/2}\left(\frac{x_{i+l,k}^a}{\sigma_{ik}^a}\right)\left(\frac{x_{j+l,k}^b}{\sigma_{jk}^b}\right);\quad \sigma_{ik}^a=\sqrt{\frac{1}{n}\sum_{l=-(n-1)/2}^{l=(n-1)/2}\left(x_{i+l,k}^a\right)^2} \qquad \text{(S12)}$$

This method is less sensitive to noise in the data and better captures subtle similarities between the datasets.

References:

1. Velichko V, Zagoruyko N: Automatic recognition of 200 words. *Int J Man-Mach Stud.* 1970, 2:223.
2. Kruskal J, Liberman M: *The symmetric time-warping problem: from continuous to discrete*. Stanford: CSLI Publications; 1999.
3. Aach J, Church GM: Aligning gene expression time series with time warping algorithms. *Bioinformatics.* 2001, 17:495-508.
4. Bar-Joseph Z, Gerber G, Simon I, Gifford DK, Jaakkola TS: Comparing the continuous representation of time-series expression profiles to identify differentially expressed genes. *Proc Natl Acad Sci U S A.* 2003, 100:10146-10151. Epub 12003 Aug 10121.
5. Clote P, Straubhaar J: Symmetric time warping, Boltzmann pair probabilities and functional genomics. *J Math Biol.* 2006, 53:135-161. Epub 2006 May 2005.
6. Tsiporkova E, Boeva V: Fusing time series expression data through hybrid aggregation and hierarchical merge. *Bioinformatics.* 2008, 24:i63-69.

Supplementary Figures

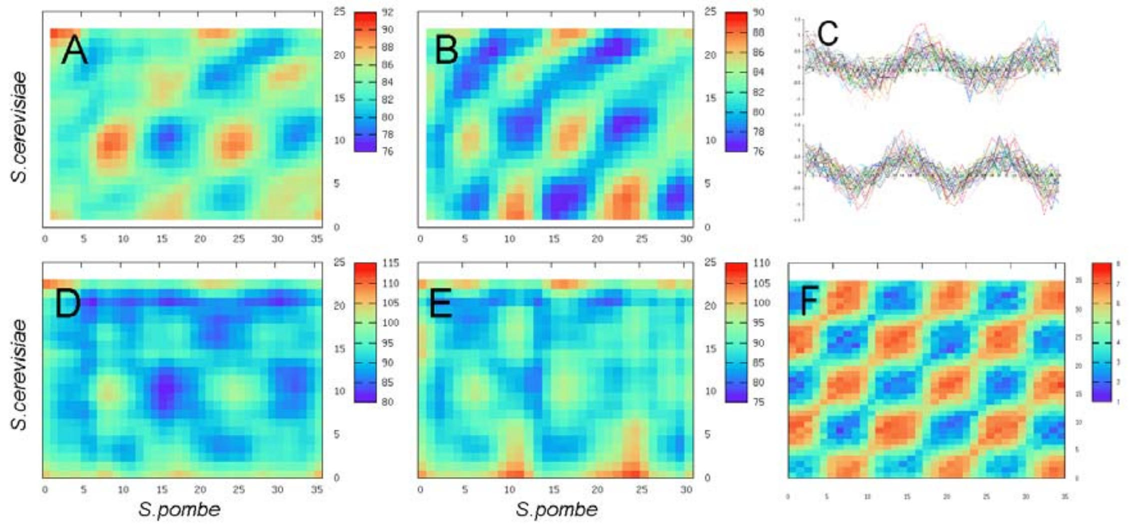

Figure. S1. Pearson and Euclidean similarity matrices

A, B Pearson similarity matrices and **D, E** Euclidean similarity matrices constructed for the selected pairs of datasets. **A**. *S.cerevisiae* cells synchronized by α-factor (Pramila et. al, 2006) versus *S.pombe* cdc25 temperature sensitive mutant (Peng et. al, 2005). **B**. *S.cerevisiae* synchronized by α-factor versus *S.pombe* synchronized by elutriation (the same authors). **C**. Two datasets simulating periodic gene expression. **F**. Pearson similarity matrix for the two simulated datasets shown in C. Colors range from blue (similar) to red (dissimilar). One can see that the Pearson matrices (A, B) are more sustainable to the noise and produce distinct valleys, resulting in smooth alignment paths (see Figure S2).

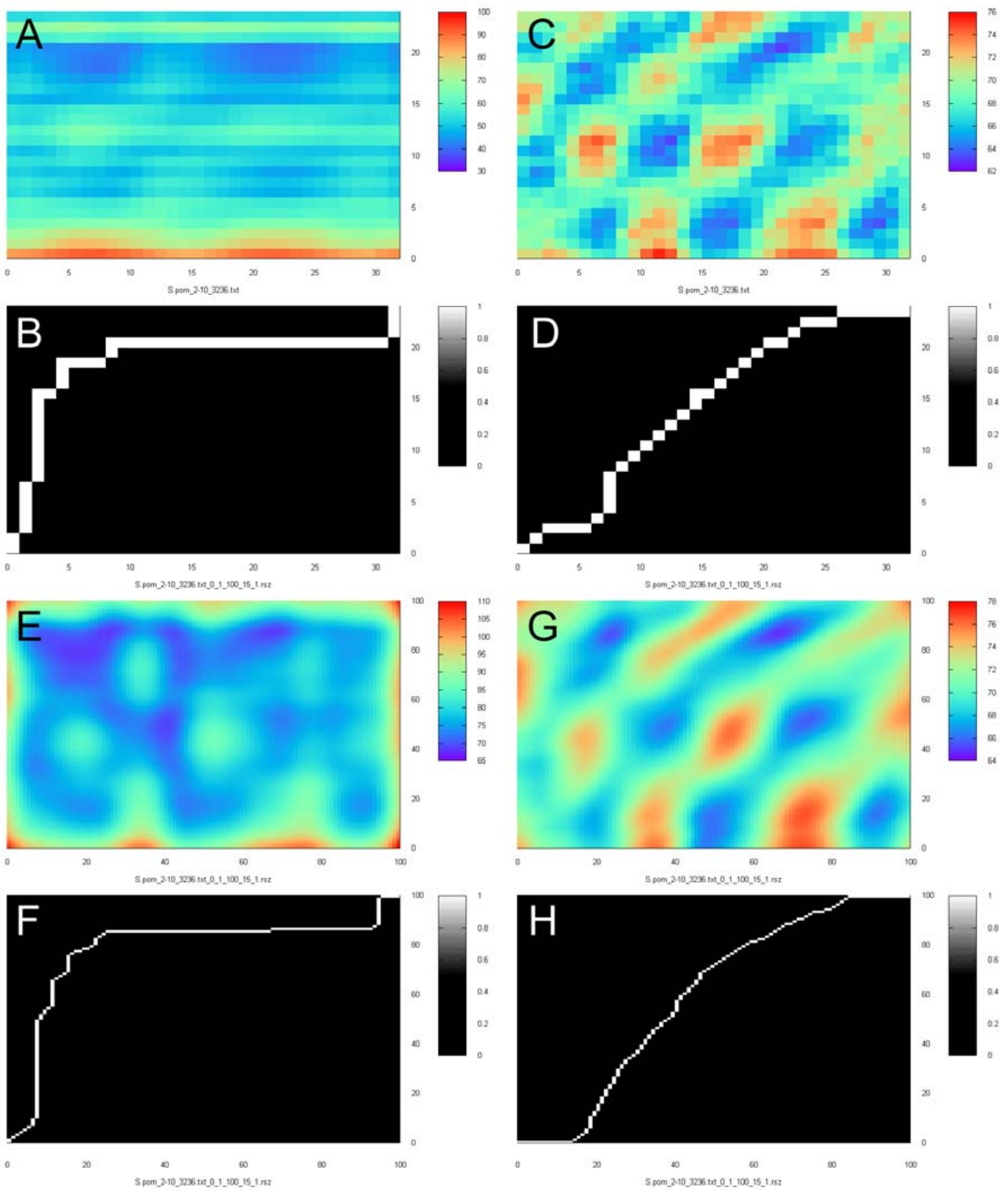

Figure. S2. Alternative alignment methods and data processing
Standard Kruscall-Liberman histogram alignment method (**A**, **B**, **E**, **F**) is compared with its modification and alternative data processing used in this study (**C**, **D**, **G**, **H**). **A**, **B** Distance matrix and alignment path for Kruscall-Liberman alignment algorithm, based on Euclidean distance matrix, no data treatment. **E**, **F** Kruscall-Liberman algorithm applied to treated (upsampling + smoothing) data. **C**, **D** Kruscall-Liberman algorithm based on Person distance matrix, no data treatment. **G**, **H** Kruscall-Liberman algorithm based on Person distance matrix applied to treated (upsampling + smoothing) data.

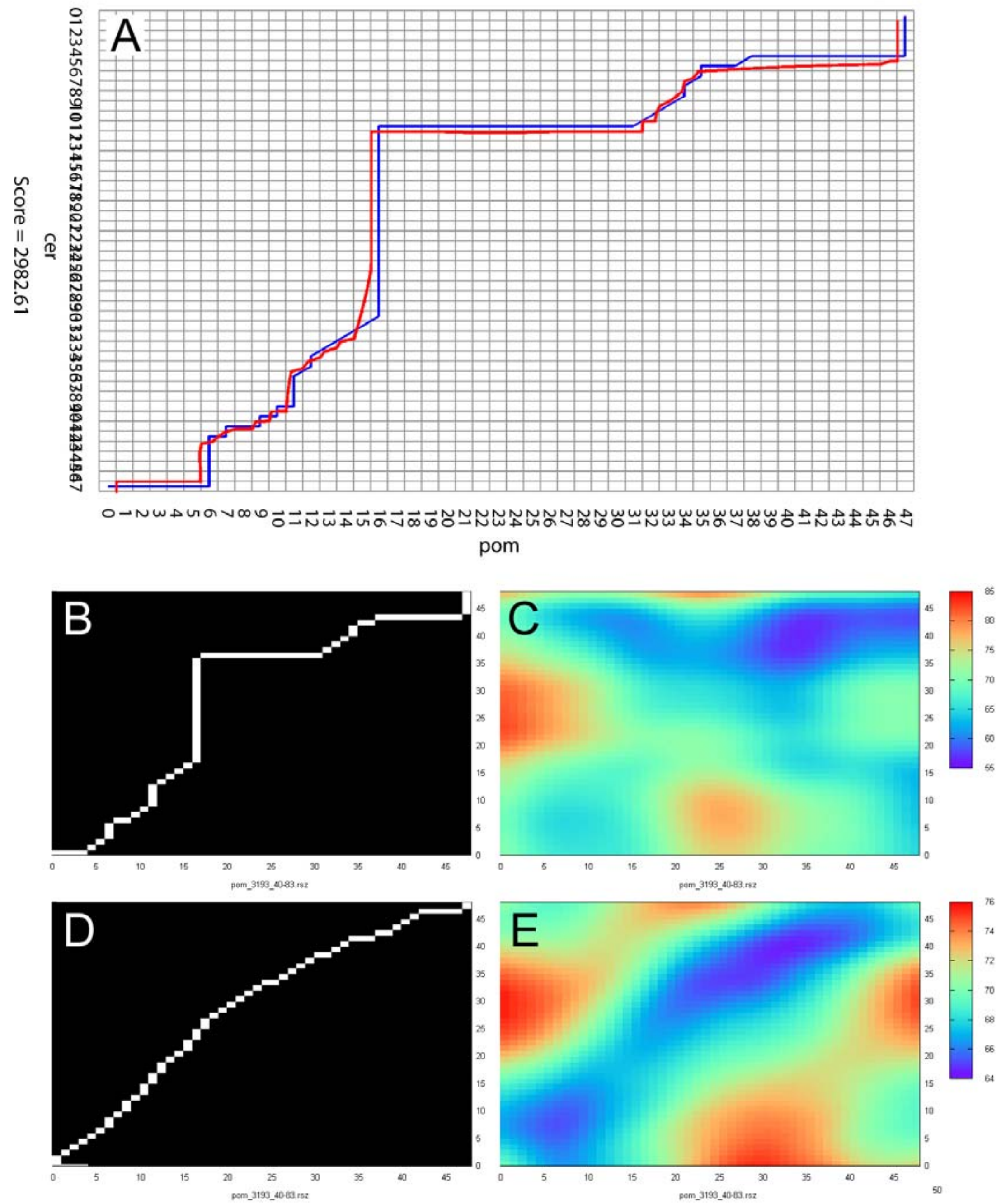

Figure. S3. Alternative methods and programs applied to a single cell cycle data
Benchmarking test comparing different methods and programs, the test was carried out on smoothed data for a single cell cycle (see Figure 1 in the main text). (A) Alignment paths generated by standard (blue line) and interpolated (red line) Kruscall-Liberman algorithm, implemented by Aach and Church (Bioinformatics. 2001, 17:495-508). (B, C) Alignment produced by program "timewarp" using standard Kruscall-Liberman algorithm. (D, E) Alignment produced by the "timewarp" using Pearson distances. Distance matrices are shown for the Euclidean (C) and the Pearson (E) methods.

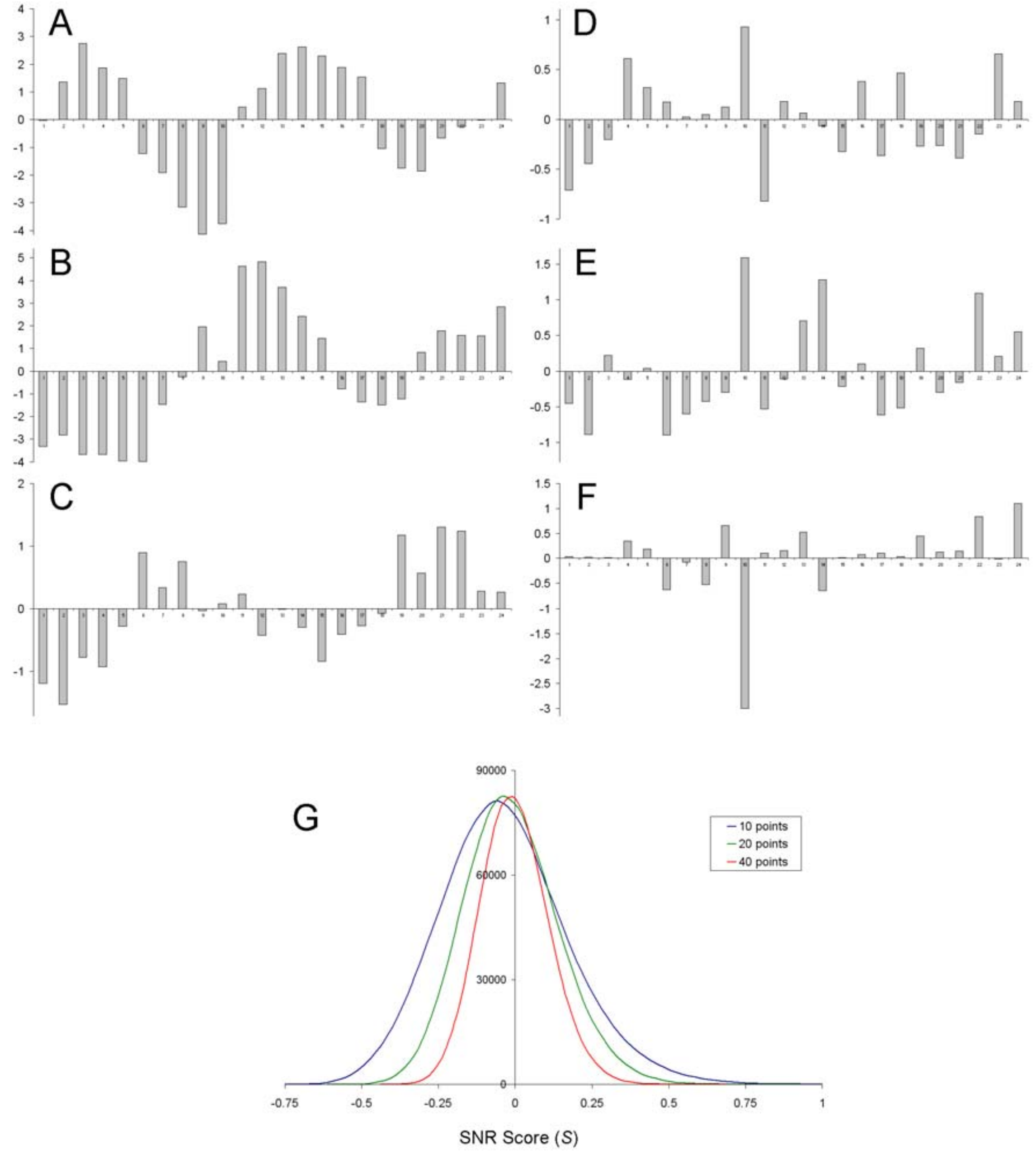

Fig. S4. Signal-to-noise ratio filtering (SNR filtering)
A-C Gene expression profiles with low noise level – difference between neighboring samples (time points) is small. **D-F** Gene expression profiles with high noise levels. Profiles generated from random values were used to build statistics for the SNR filtering. **G** shows null noise models (distributions of the noise score *S*), constructed from 10^6 random profiles each for various numbers of samples in a single profile.

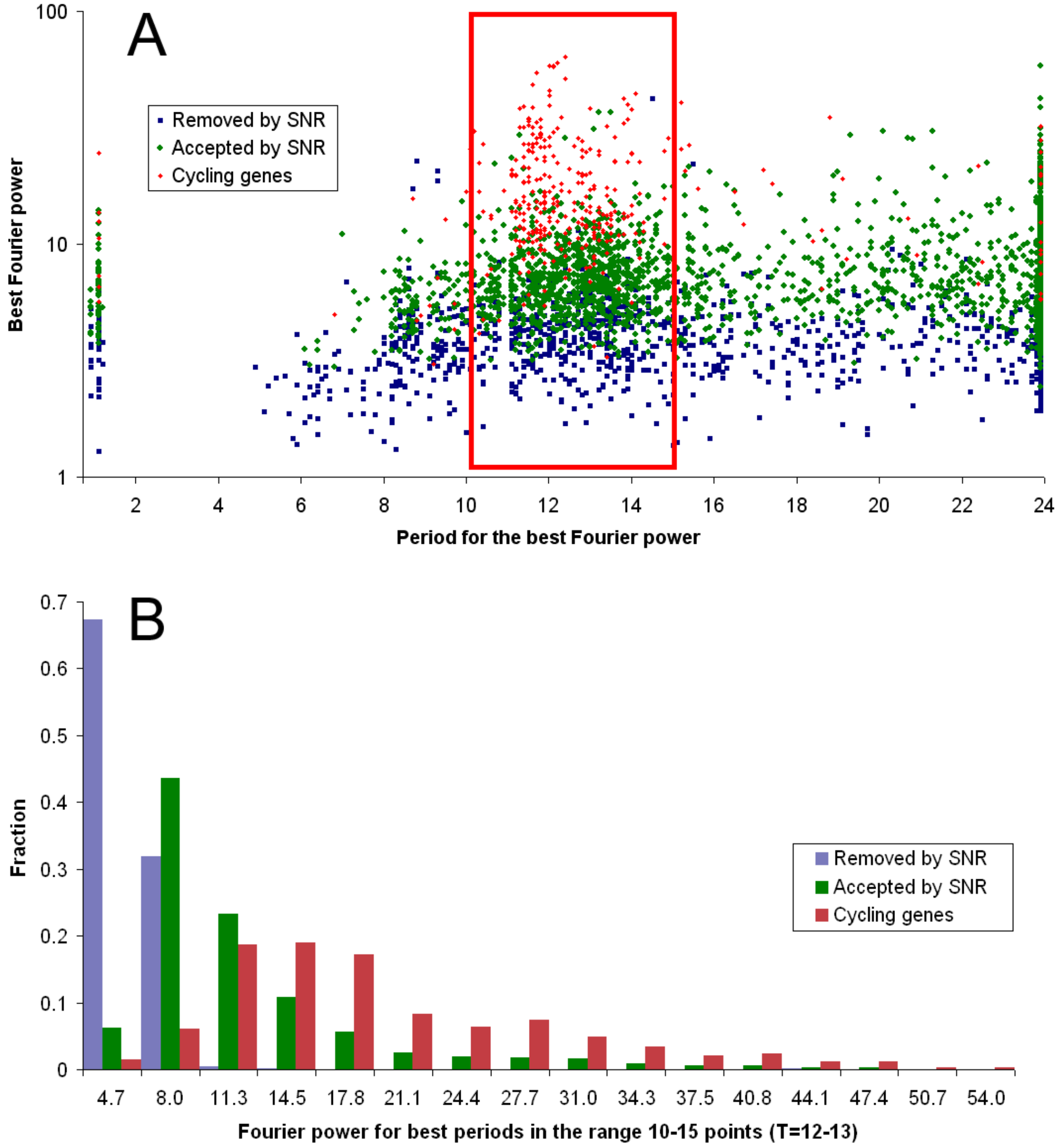

Figure. S5. Periodically expressed genes and SNR filtering
A. Scatter plot shows dependence of the peak power from the peak period for expression profiles in the analyzed *S.cerevisiae* dataset. Red box marks the area of the expected periodicity for the given dataset. Cycling genes are the 500 profiles reported in the previous studies. One can see that SNR removes genes with the poor Fourier peak scores. **B.** Distributions of the best Fourier power for the same three categories of genes, the distributions are given for the genes within the red box area on A. Red (cycling + passed SNR) and green (passed SNR) profiles were accepted for the construction of alignment. Profiles marked by blue were eliminated from the further consideration as

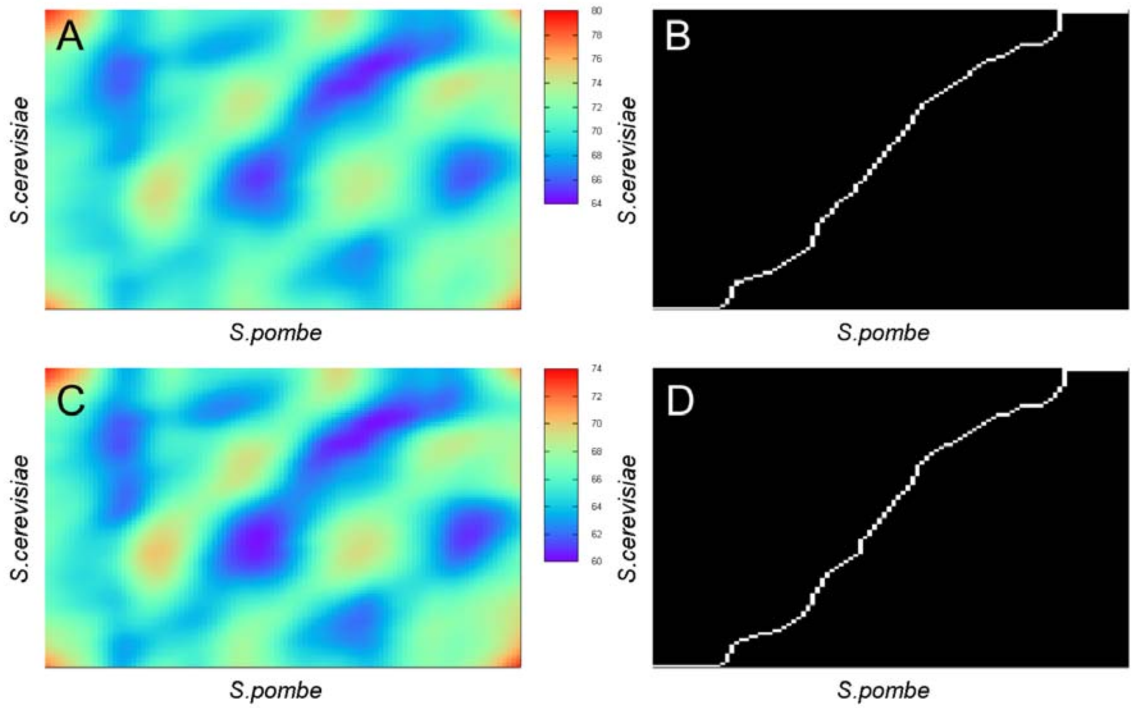

Figure S6. Profiles for 500 best cycling genes have no effect on time warping
A Pearson distance matrix and **B** global alignment for the entire datasets of 3193 profile pairs. **C** Pearson distance matrix and **D** global alignment for the same dataset, after removal of the 500 best cycling genes, identified for *S.cerevisiae* in the previous studies.

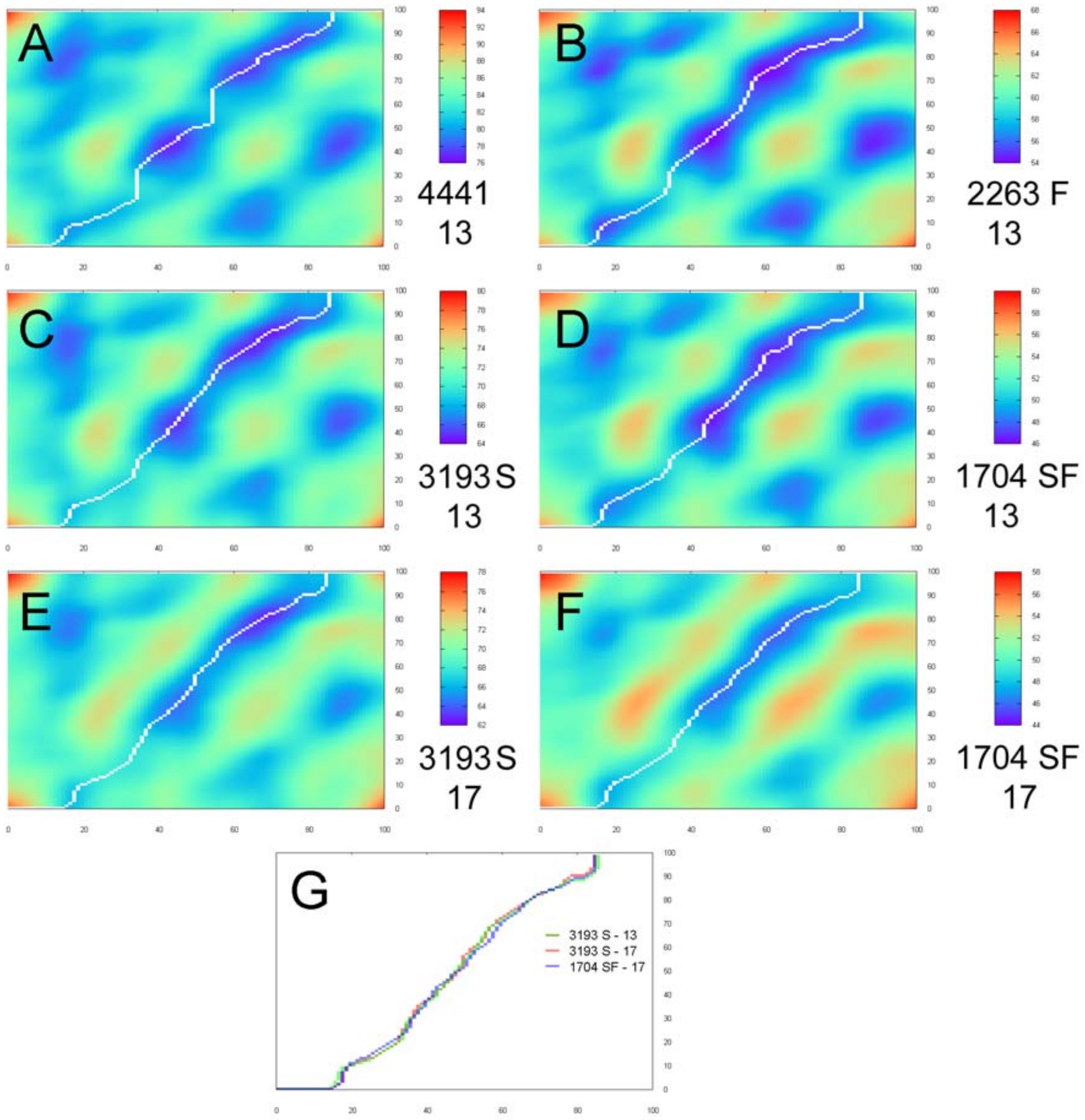

Fig. S7. Data treatment and quality of alignments
A. Pearson similarity matrix and the alignment path (white line) for all 4441 orthologous profile pairs from the unfiltered datasets. **B.** Effect of Fourier filtering of the initial datasets, 2263 profile pairs remained (see also Figure S6). **C.** Effect of SNR filtering alone, 3193 profile pairs. This SNR filtering was accepted for use in global alignment. *D*. Combined effect of Fourier and SNR filtering, 1704 profile pairs remained. *C*, *E*, *D*, *F*. Effects of window size (*n*, see Methods) at the matrix construction step. Larger windows in *E* and *F* (n=13 in *C*, *D*; n=17 in *E*, *F*) result in more distinct and narrow valleys (compare *D* and *F*). *G*. Superposition of the alignment paths from *C*, *E* and *F* shows essentially the same global path, suggesting robustness of alignment. All datasets were normalized, upsampled and smoothed prior to the matrix construction. SNR filtering was performed before upsampling and smoothing, Fourier filtering was performed after the upsampling/smoothing step.

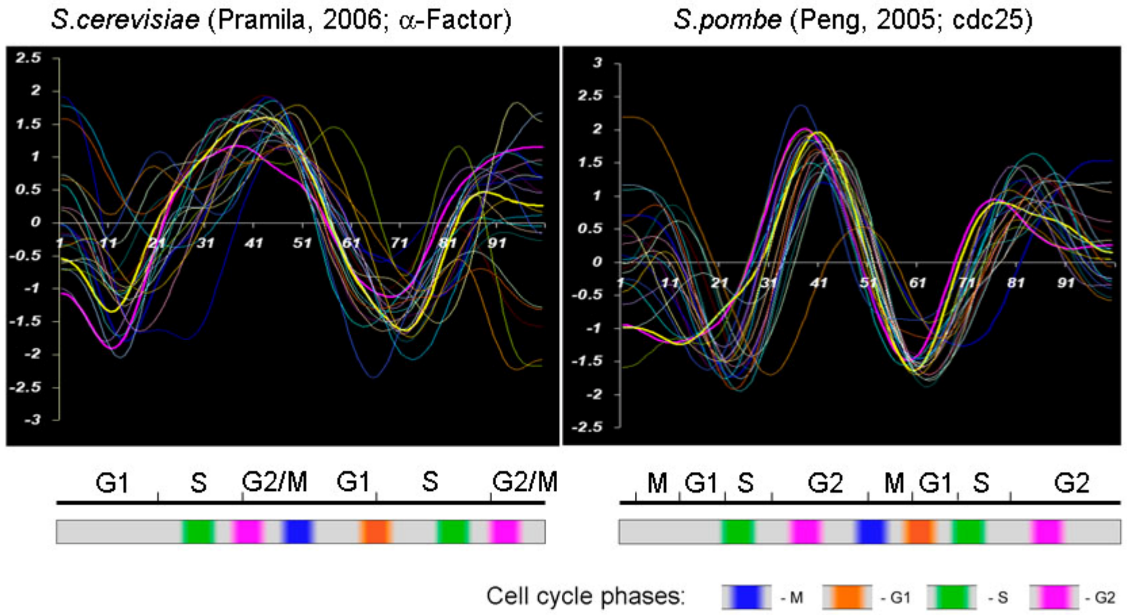

Fig. S8. Concordant expression of genes involved in ribosomal biogenesis
Two panels (left for *S.cerevisiae* and right for *S.pombe*) represent expression profiles of representative genes involved in ribosomal biogenesis. *S.cerevisiae*, left panel - *SPAC31A2.07C, SPBC543.06C, SPAC2G11.11C, SPBC3D6.12, SPCC330.09, SPAC1F7.02C, SPAC19D5.05C, SPAC19A8.07C, SPBP22H7.02C, SPBC11G11.03, SPAC1B3.13, SPAC3F10.16C, SPCC4G3.18, SPAC16C9.03, SPAC6F6.03C, SPBC16E9.10C, SPBC1604.09C, SPAC22A12.12C, SPAC2F7.05C, SPBC800.06, SPAC13G7.08C, SPCC16C4.08C* and their orthologs in *S.pombe*, right panel -*YDL031W, YHR169W, YKL078W, YLR129W, YGR145W, YMR290C, YHR148W, YNL075W, YPR112C, YKL009W, YPL126W, YGL099W, YHR197W, YHR170W, YNR053C, YLL034C, YOL080C, YOL142W, YPR041W, YOL077C, YNL182C, YKL021C*. Diagrams below the panels represent cell cycle phases as established by authors of original datasets (Pramila, 2006; Peng, 2005) using FACS analysis. Color diagrams below represent approximate layout of cell cycle phases inferred from expression of cell cycle markers in this study.

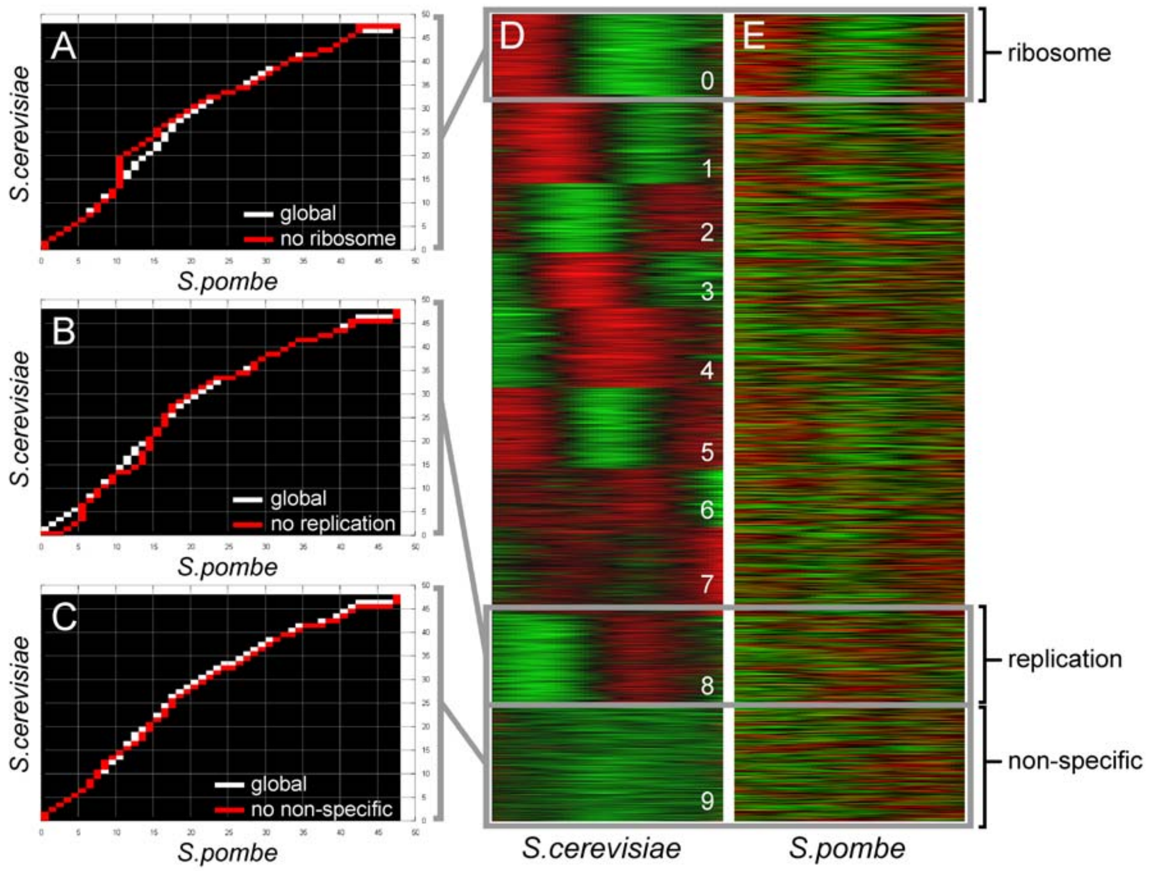

Fig. S9. Contribution of functional gene groups to the alignment quality
A-C alignment paths after removal of a certain classes of profiles (the paths are in red) in comparison with the global alignment path (white line). **D.** K-means clustering of *S.cerevisiae* expression data, selected profile classes are shown in boxes. The orthologous profiles from *S.pombe* are shown in panel *E*. Removal of any single class of profiles does not change the global alignment path dramatically, suggesting that there is no single "core" group of profiles, which determines the global alignment path.

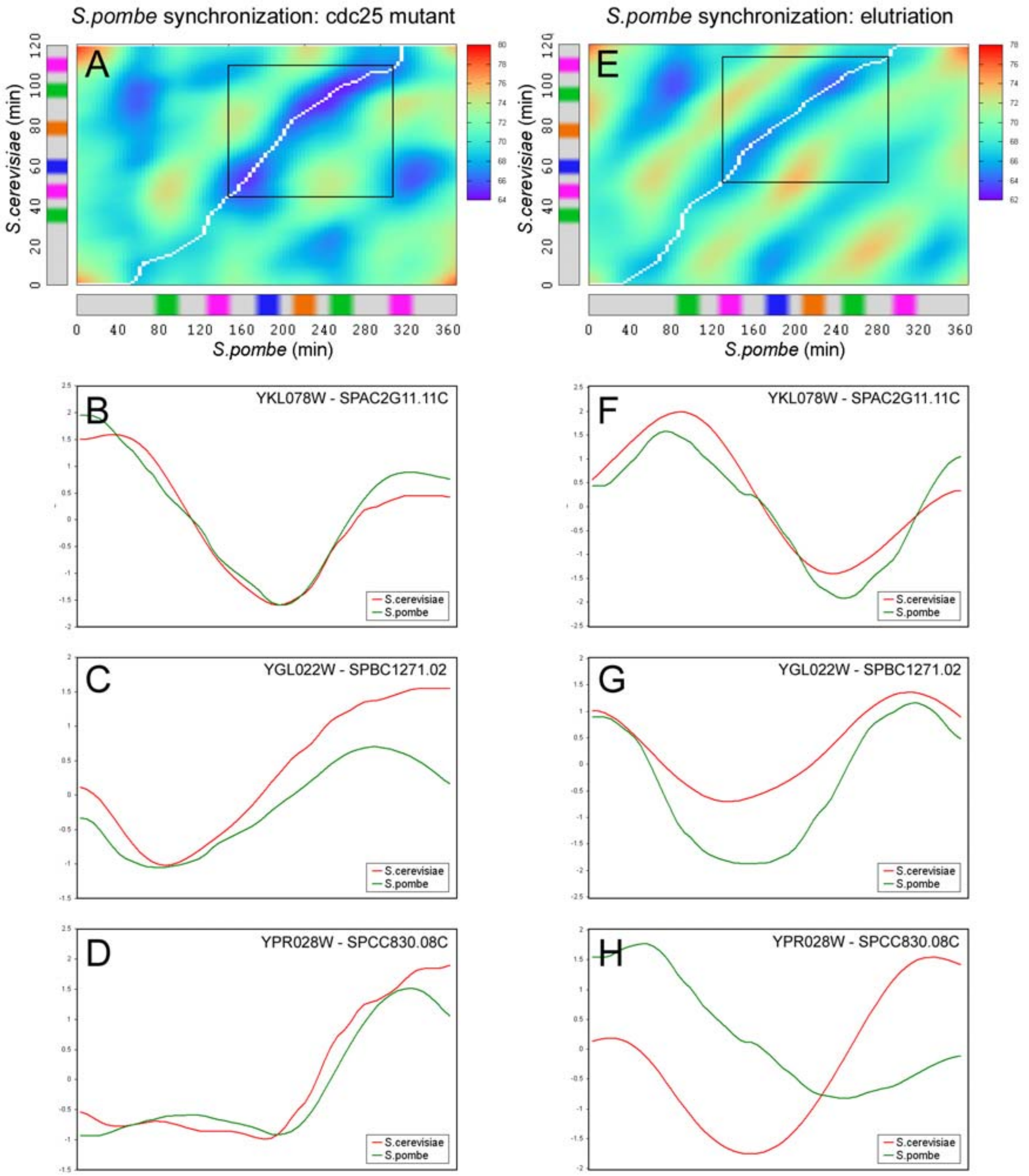

Fig. S10. Effect of selected method for cell synchronization

Pearson distance matrices, alignment paths and orthologous profiles are shown for two pairs of datasets, in which *S.cerevisiae* cells were synchronized using α-factor (replicates) and *S.pombe* cells were synchronized using either cdc25 temperature sensitive mutant (**A**-**D**) or elutriation (**E**-**H**). The expression profiles are given for a single cell cycle (shown by black boxes in **A** and **E**) from the aligned data sets. Profile pairs corresponding to some of the best concordant genes were selected from the first data set (cdc25, **B**-**D**). The same profile pairs were then retrieved from the second dataset (elutriation, **F**-**H**). While the majority of genes are method insensitive, some show dependence from the method of synchronization (*S.pombe* profile in **D** and **H**; see also Table S2).

Time Cluster	N	Sub-cluster	N	GO Name	GO Type-ID	In time cluster	In sub-cluster	P-value
0	365	2	33	mitochondrial respiratory chain	C-5746	5	4	0.02
				mitochondrion	C-5739	73	18	0.09
				oxidoreductase activity	F-16491	19	8	0.09
				aerobic respiration	P-9060	9	5	0.11
		3	39	ribosome biogenesis and assembly	P-42254	11	6	0.15
				cell differentiation	P-30154	25	9	0.21
				rRNA metabolic process	P-16072	4	3	0.37
		5	37	ribosome	C-5840	15	8	0.01
				small ribosomal subunit	C-15935	5	4	0.04
				translation	P-6412	24	9	0.20
5	271	0	33	mitochondrial ribosome	C-5761	7	6	0.00
				mitochondrial lumen	C-31980	16	9	0.00
				mitochondrial part	C-44429	30	12	0.01
				translation	P-6412	19	9	0.01
		3	33	cytoskeletal protein binding	F-8092	4	4	0.03
				cell cortex	C-5938	3	3	0.18
		7	33	ribosome biogenesis and assembly	P-42254	14	11	0.00
				nucleolus	C-5730	9	8	0.00
				rRNA processing	P-6364	5	5	0.00
				nuclear lumen	C-31981	24	12	0.00
9	518	2	52	response to unfolded protein	P-6986	7	5	0.03
				response to biotic stimulus	P-9607	9	5	0.21
		3	73	DNA replication	P-6260	16	10	0.00
				DNA strand elongation	P-22616	8	6	0.01
				replication fork	C-5657	7	5	0.08
		4	51	SAGA complex	C-124	4	3	0.38
		5	60	RNA elongation	P-6354	5	4	0.23
		6	43	cellular lipid catabolic process	P-44242	4	3	0.07
				nucleosome assembly	P-6334	7	4	0.08
		9	89	rRNA processing	P-6364	46	34	0.00
				ribosome biogenesis and assembly	P-42254	74	42	0.00
				RNA binding	F-3723	50	24	0.00
				35S primary transcript processing	P-6365	22	14	0.00
9	518	-	-	ribosome biogenesis and assembly	P-42254	200	74	0.00
				rRNA processing	P-6364	114	46	0.00
				nucleolus	C-5730	137	52	0.00
				mitochondrial part	C-44429	212	23	0.23
				acetylglucosaminyltransferase	F-8375	4	4	0.41
				ribosome assembly	P-42255	36	17	0.41
				carboxylic acid transporter activity	F-46943	10	7	0.45
				35S primary transcript processing	P-6365	52	22	0.45
				nucleosome	C-786	8	6	0.48

Table S1. Distribution of GO terms among time clusters

Results of clustering using individual alignment paths are shown. Presence of GO terms and significance of GO terms distributions among the clusters was established using GenMAPP program. The First column contains number of time clusters, shown in Figure 3, the second column is the number of profiles in the time clusters. The third column is the number of sub-clusters in each time cluster and the fourth column is the number of profiles in each sub-cluster. The last columns show the GO terms enrichment in sub-clusters (and in time cluster 9, last 10 rows) and the corresponding p-values.

S.cerevisiae	*S.pombe*	*r* (S.p, cdc25)	*r* (S.p, elutriation)
YGL022W	SPBC1271.02	0.98	0.98
YKL078W	SPAC2G11.11C	0.98	0.95
YLR398C	SPCC550.03C	0.99	0.90
YCR072C	SPCC18.05C	0.99	0.85
YNL263C	SPBC25H2.06C	0.97	0.76
YER082C	SPAC959.03C	0.99	0.30
YBR068C	SPAC869.10C	0.98	0.13
YAR071W	SPBC428.03C	0.98	0.10
YPR028W	SPCC830.08C	0.98	-0.15
YDR447C	SPCC24B10.09	0.98	-0.84

Table S2. Consistency between two selected methods of cell synchronization

Table shows agreement (correlation, third column) for 10 best concordant orthologous profile pairs, selected from one aligned dataset *S.cerevisiae* (synchronized using α-factor) - *S.pombe* (synchronized using cdc25). Agreement for the same orthologous profile pairs (the last column) was measured in another aligned dataset, in which a technical replicate was taken for *S.cerevisiae* (synchronized using α-factor) and a microarray data obtained using yet another technique was taken for *S.pombe* (synchronized using elutriation). In the case of second alignment, the majority of the previously selected profile pairs display positive correlation, while some show poor agreement (see also Figure S10).
